# Directional Multi-Resonant Micro-Electromechanical System Acoustic Sensor for Low Frequency Detection

**DOI:** 10.3390/s24092908

**Published:** 2024-05-02

**Authors:** Justin Ivancic, Fabio Alves

**Affiliations:** Department of Physics, Naval Postgraduate School, Monterey, CA 93943, USA

**Keywords:** MEMS acoustic sensor, multi-resonant acoustic sensor, directional acoustic sensor, underwater acoustic sensor

## Abstract

This paper reports on the design, modeling, and characterization of a multi-resonant, directional, MEMS acoustic sensor. The design builds on previous resonant MEMS sensor designs to broaden the sensor’s usable bandwidth while maintaining a high signal-to-noise ratio (SNR). These improvements make the sensor more attractive for detecting and tracking sound sources with acoustic signatures that are broader than discrete tones. In-air sensor characterization was conducted in an anechoic chamber. The sensor was characterized underwater in a semi-anechoic pool and in a standing wave tube. The sensor demonstrated a cosine-like directionality, a maximum acoustic sensitivity of 47.6 V/Pa, and a maximum SNR of 88.6 dB, for 1 Pa sound pressure, over the bandwidth of the sensor circuitry (100 Hz–3 kHz). The presented design represents a significant improvement in sensor performance compared to similar resonant MEMS sensor designs. Increasing the sensitivity of a single-resonator design is typically associated with a decrease in bandwidth. This multi-resonant design overcomes that limitation.

## 1. Introduction

The design, modeling, and analysis of a multi-resonant, directional, micro-electromechanical system (MEMS) acoustic sensor is presented. Decades of research and development have been dedicated to better understanding microscale, directional acoustic devices. These small device designs are useful for creating small acoustic vector sensors (AVS), which are capable of determining the direction of arrival (DOA) of incoming sound [1,2]. MEMS devices are popular for use in acoustics because they allow detectors to be small, lightweight, and have low power consumption requirements. They are ideal for creating manually portable AVS systems. The motivation of this research is to improve upon existing MEMS resonant acoustic sensors with a multi-resonant design that increases the frequency bandwidth of the sensor while maintaining a high signal-to-noise ratio (SNR) and preserving directionality characteristics.

### 1.1. Subwavelength-Sized Directional Sensors

Maintaining the sensor’s cosine-like directionality is a key factor in this research. Directional sensors have an acoustic sensitivity that varies with the sound’s DOA. Frequently, although not necessarily, microscale acoustic sensors have a dipole (or cosine-like) directionality, where the maximum sensitivity is exhibited when the acoustic wave travels normally in relation to the face of the sensor. The sensitivity decreases, like a cosine, to zero when the wave direction is rotated 90 degrees so that it propagates parallel to the sensor face. This effect is due to the gradient that is formed by the incident sound pressure on the front and back of the sensor. The presented sensor has just such a cosine-like directionality. Understanding the directionality of an acoustic sensor is necessary to determine the acoustic DOA. Some other microscale sensor designs display different directionality patterns and are discussed in this section.

In 2018, Zhou and Miles [3] demonstrated an acoustic flow detector using nanofibers. The nanofibers were driven by viscous forces, created by the particle motion of the surrounding medium of the sensor when subjected to an acoustic wave. This design demonstrated a dipole directionality and a flat sensitivity curve of zero dB over a wide frequency range (100 Hz to 10 kHz).

Research presented by Lee et al. [4] in 2020 demonstrated how a sensor consisting of coupled Helmholtz resonators could be used for DOA determination. Two designs were presented, a dual resonator and a triple resonator, each with their own directionalities. By comparing the pressure response of the resonator chambers, the DOA of an acoustic source could be determined. The triple resonator design demonstrated a 360-degree DOA coverage.

In 2022, Chen et al. [5] presented an acoustic detector consisting of a four-sided Helmholtz resonator placed in the center of an array of phononic crystal cylinders. The sensor design demonstrated a cross-shaped directionality, with the maximum sensitivities being 90 degrees apart from each other. The design showed a 280:1 gain in acoustic pressure at resonance.

Also in 2022, Chen et al. [6] presented a gradient acoustic metamaterial coupled with a space-coiling structure acoustic device consisting of an array of metamaterial plates that incrementally increased in size. The design exploited wave compression effects to amplify the sound signal. The sensor has a unique directionality, with one large lobe at the front of the array and a small back lobe. Two of these sensors were aligned in a canted configuration to determine the acoustic DOA. Despite being small compared to acoustic wavelengths, many of these directional acoustic sensors are significantly larger than MEMS sensors.

### 1.2. Resonant MEMS Sensors

This research is interested in acoustic sensors that are capable of detecting quiet or distant acoustic sources. Operating the sensor at resonance helps achieve this goal. Typical microphones are designed to operate at frequencies that are far from their resonances so that they maintain a constant sensitivity over a large frequency range [7]. However, to maximize the acoustic sensitivity, achieve a high SNR, or mechanically filter unwanted acoustic noise, it is advantageous to operate acoustic detectors at or near resonance. One common MEMS acoustic sensor design consists of a vibrating cantilever beam or paddle connected to a substrate. Research has been steadily conducted on this kind of MEMS acoustic sensor.

In 2019, Rahaman and Kim [8] presented a disc-shaped double-wing MEMS acoustic sensor with a dipole directionality. The sensor utilized a piezoelectric sensing system. An AVS was constructed using two of these sensors. This AVS demonstrated an ability to calculate the acoustic DOA over a 90-degree arc. In 2020, Rahaman and Kim [9,10] presented a different double-wing sensor with rectangular wings. The sensor demonstrated a cosine-like directionality. The reported sensitivity was 3.45 mV/Pa (−49.2 dB re 1 V/Pa) at 1 kHz, with an SNR of approximately 68.5 dB. An array of three of these sensors was used to localize a sound source.

In 2020, Espinoza et al. [11] demonstrated two MEMS acoustic sensors: a double-wing design and a cantilever paddle design. These sensors were intended for use underwater by placing them in a silicone oil-filled housing. The housing was then submerged in water. The paddle and double-wing sensors demonstrated a peak sensitivity of approximately 5.5 mV/Pa and 6 mV/Pa (−45.2 dB and −44.4 dB re 1 V/Pa), respectively, at resonance. When operating in air, both sensors demonstrated a cosine-like directionality pattern; however, in water, the directionality pattern was distorted with unequal lobe sizes.

In 2020, Rabelo et al. [12] presented a double-wing design with a closed cavity behind the sensor. This configuration allowed for comparable rocking and bending modes. The acoustic DOA was demonstrated to be proportional to the phase shift between these two modes. This allowed for DOA determination, using a single sensor, over a 180-degree arc with an accuracy of 3 degrees. The sensitivity of the sensor was determined to be on the order of 1 V/Pa (0 dB re 1 V/Pa).

In 2021, Li et al. [13] presented methods to optimize the dimensions of a piezoelectric MEMS cantilever beam acoustic sensor. The peak sensitivity of the sensor at resonance (30 kHz) was 148 V/m/s.

In 2022, Li et al. [14] followed up their work of improving the piezoelectric MEMS acoustic sensor’s bandwidth and sensitivity performance. They created an array of identical cantilever beams and optimized the layer thickness of the devices. A single cantilever sensor demonstrated a peak sensitivity of approximately 1 V/m/s with a narrow resonance peak at 48.7 kHz. An array of 210 cantilevers with identical designs improved the sensitivity to 2 V/m/s. The bandwidth also increased in frequency range. The sensitivity was essentially constant from 44.9 to 48.9 kHz. This work demonstrated how an array of beams can improve performance by broadening the response through multiple resonances.

In 2022, Rahaman and Kim [15] presented an AVS made from an array of three double-wing, resonant, MEMS acoustic sensors. The maximum sensitivity of the sensors was approximately 100 mV/Pa (−20 dB re 1 V/Pa) at the bending resonant mode (11.9 kHz). The AVS demonstrated 360 degrees of coverage in azimuth and elevation, but one required a priori information of the other.

In 2023, Ivancic et al. [16] demonstrated a symmetric double-wing design that emphasized the bending mode. The sensor demonstrated a sensitivity of 59 V/Pa (35.4 dB re 1 V/Pa) and an SNR of 88 dB at 1 Pa over the bandwidth of the sensor circuity. The sensor demonstrated a cosine-like directionality in air and a distorted cosine directionality in water (similar to [11]). An AVS was assembled, which consisted of two of these sensors and a commercial omnidirectional acoustic sensor. The AVS demonstrated a 360-degree DOA coverage with a 3.5-degree accuracy.

### 1.3. Multi-Resonant MEMS Sensors

A limitation with many resonant sensor designs is that they operate in a narrow frequency band, which makes the sensors less effective for detecting broadband acoustic sources. This research is interested in broadening that frequency band. As suggested by [14], combining multiple vibrating wings into a single sensor can be an effective way to broaden the frequency band of the sensor.

Multi-resonant MEMS acoustic sensors employ multiple resonators with differing resonant frequencies. This increases the overall bandwidth of the sensor. In 2013, Baumgartel et al. [17] presented a multi-resonant MEMS acoustic sensor that consisted of thirteen cantilevered paddles with a piezoelectric vibration sensing scheme. The resonant frequencies of each paddle varied from 860 Hz to 6.2 kHz, with a maximum sensitivity of 202.6 mV/Pa (−13.9 dB re 1 V/Pa). The sensitivity of the sensor remained above 2.5 mV/Pa (−52.0 dB re 1 V/Pa) over the designed frequency range of the sensor (240 Hz to 6.5 kHz). In 2015, Shkel et al. [18] followed up this research with thirteen cantilevered paddle designs, using the resonant frequencies of each paddle to mechanically isolate sound (human speech) from noisy background environments. They demonstrated that the sensor could improve automated speech recognition by 62.7% from a signal with a 15 dB SNR.

In 2020, Liu et al. [19] presented two piezoelectric MEMS cantilevered paddle arrays, one with ten paddles and the other with nine. The resonant frequencies of the ten-paddle and nine-paddle arrays ranged from 856 to 4889 Hz and 5380 to 8820 Hz, respectively. Using these arrays in conjunction demonstrated an improvement in SNR for typical human speech frequencies. The maximum acoustic sensitivity was 202.1 mV/Pa (−13.9 dB re 1 V/Pa) at 856 Hz.

In 2021, Kang et al. [20] demonstrated an MEMS acoustic device, inspired by the human cochlea, consisting of sixteen cantilever beams. The cantilevers were of different sizes and operated over multiple bending modes of each beam. The beams were designed so that the entire frequency band of the sensor was covered by one or more of these modes. The sensor demonstrated a sensitivity of approximately 71 mV/Pa (−23 dB re 1 V/Pa) over a frequency range of 300 Hz to 8 kHz. The sensor demonstrated a cosine-like directionality.

In 2022, Alves et al. [21] presented a double-wing design where the torsional legs were offset from the center of the bridge that connected the wings. This configuration created two separate bending mode resonances (one for each wing). This allowed for a wider resonance bandwidth when the responses of each wing were combined. The sensor demonstrated a 13 V/Pa (22.3 dB re 1 V/Pa) maximum sensitivity with a 91 dB SNR. The sensor demonstrated a cosine-like directionality in air.

The resonant sensors discussed above provide high sensitivity and directionality but are limited in effective bandwidth. Most of the multi-resonant sensors demonstrated broader bandwidths but lacked high acoustic sensitivities. The sensor presented in this paper combines a high sensitivity with a broader bandwidth. It utilizes a wing design inspired by those described in [16]. However, instead of consisting of two mechanically coupled identical wings, this design consists of six independent wings. Each wing has a different resonant frequency so that the sensor has increased bandwidth while maintaining a high sensitivity and SNR across that bandwidth.

### 1.4. Environmental Sensing

The multi-resonant MEMS acoustic sensor presented in this paper is ideal for use in AVS designs. The acoustic sources of interest to this research are gunshots, drones, and underwater vehicles. However, this sensor design can be modified to detect and monitor a variety of sound sources (e.g., road vehicle noise, airborne noise, environmental noise) in a wide range of acoustic environments. While a single AVS can provide a bearing to a sound source, a distribution of these AVSs (alone or as part of a larger suite of sensors) can provide the ability to determine a sound source’s location.

## 2. Design and Modeling

### 2.1. Design Requirements

The MEMS sensor was microfabricated out of a 400 μm thick silicon-on-insulator (SOI) wafer with a 25 μm device layer. The vibrating wings were etched into the device layer. Likewise, the substrate below the wing was etched all the way through to allow the wing to vibrate freely. Gold pads were deposited onto the device layer to provide ohmic contact. Insulating trenches were etched onto the device layer to electrically separate the vibrating wing from the fixed substrate. The sensor was fabricated by the MEMSCAP [22] commercial foundry.

**Figure 1:**
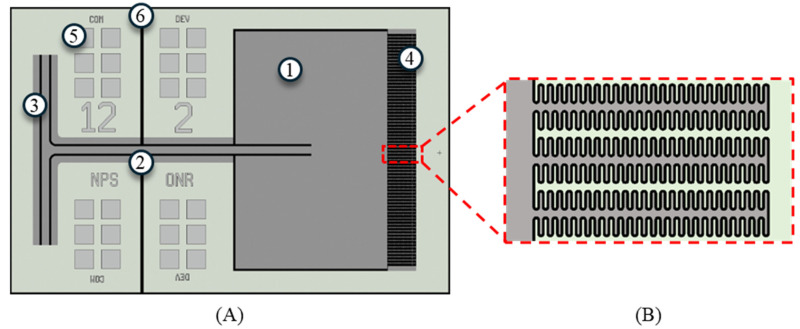
Layout of single wing of the sensor design. (**A**) Layout of entire wing: (1) wing, (2) bridge, (3) torsional leg, (4) comb fingers, (5) gold wire bonding pad, and (6) a groove in the device layer electrically separating the wing from the substrate. (**B**) Zoomed-in view of fishbone shape of comb fingers. The dark grey areas under the wing and surrounding the bridge and torsional legs represent a trench that passes through the base layer of the sensor.

Individual resonators in the array consist of a vibrating wing connected to a substrate via a bridge and torsional legs, as shown in Figure 1. When exposed to sound waves, the wings vibrate normally to the plane of the substrate. At the end of each wing, fishbone-style comb fingers are interlaced with corresponding comb fingers on the substrate. When the wing vibrates, the capacitance between the wing and substrate varies with the deflection of the wing. The sensor is cemented into an open cavity in a printed circuit board (PCB) and wire-bonded to a circuit that converts the sensor capacitance to an output voltage. Similar capacitive sensing schemes are described in more detail in [12,23].

The resonance frequency of a wing (or paddle)-shaped MEMS acoustic sensor is, in part, a function of the physical parameters of the wing and bridge (e.g., wing size, bridge length, layer thickness, material). The sensor parameters were selected to align each wing to different desired resonant frequencies.

While a sensor with a high quality factor is good for detecting a specific tone, it can limit the detection of broader acoustic sources or tones outside the passband [16]. One promising way to overcome these limitations is to use multiple resonators with near resonances to broaden the response [14]. To explore this idea, two similar multi-resonant sensors (versions V11 and V12) were produced. These designs operated nearly identically, except for slightly shifted resonant frequencies.

The design criteria was to support detection of sound from 300 to 500 Hz while maintaining a high sensitivity and SNR across the entire sensor bandwidth. The target SNR of the overall sensor should be comparable to the SNRs of the individual resonators. Additionally, the sensor should demonstrate a cosine-like directionality. The design was also constrained by manufacturing limitations (foundry design rules) [22].

**Figure 2:**
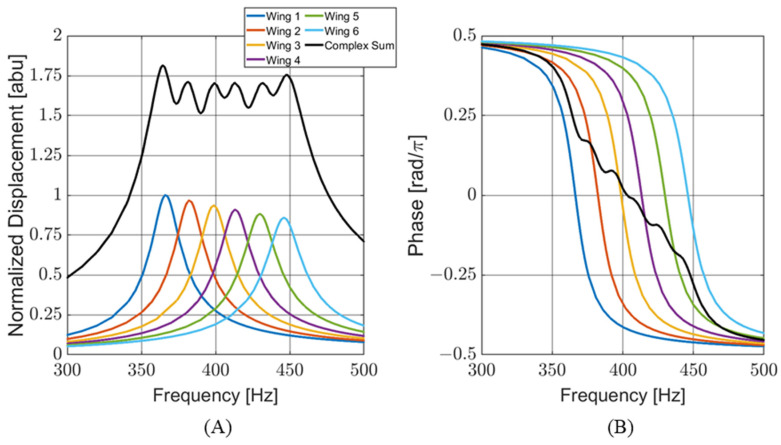
Computer-modeled behavior of sensor: (**A**) normalized wing displacement for individual wings and the complex sum of the all the wings; (**B**) individual wing phases and complex sum. The phases are offset so that the phase equals zero at resonance.

To meet these criteria, a sensor consisting of six individual wings was conceptualized. Each wing was designed with a different resonant frequency to cover the target bandwidth. The response of the wings to the incoming sound was transduced in capacitance and linearly correlated with the vibration. The output of each wing was wire-bonded to the same port on the capacitive readout circuitry. This configuration places the capacitors of each wing in parallel, creating a single sensor output that can be modeled as the complex sum of the outputs of the individual wings. Figure 2A shows a finite element (FE) simulation of the frequency response for individual wing displacements and the complex addition of all the wings for sensor design V11. The graph is normalized to the maximum displacement of wing number 1. Figure 2B shows the phase response of each wing (with respect to a driving acoustic signal) during a frequency sweep. At resonance, each wing behaves like a harmonic oscillator. However, the phase response of the whole sensor is more complex than that of a single wing.

### 2.2. Design Parameters

The sensor wings used in this design differ from similar paddle designs. For the purposes of this paper, a paddle design consists of a vibrating paddle that is directly connected to a substrate via a bridge. The bridge acts as a fixed cantilever. In the wing design, the bridge connects to torsional legs. The torsional legs then connect to the substrate and twist while the bridge bends. There are two primary reasons for including the torsional legs in this design. First, the torsional legs allow the resonance frequency of the wing to be lowered while still meeting size and manufacturing limitations. Second, our previous investigations into paddle designs revealed that the cantilever connection between the beam and substrate was structurally weak and prone to failure. Designs that include torsional legs reduce the stress on the pivot points and are less prone to failure.

**Figure 3:**
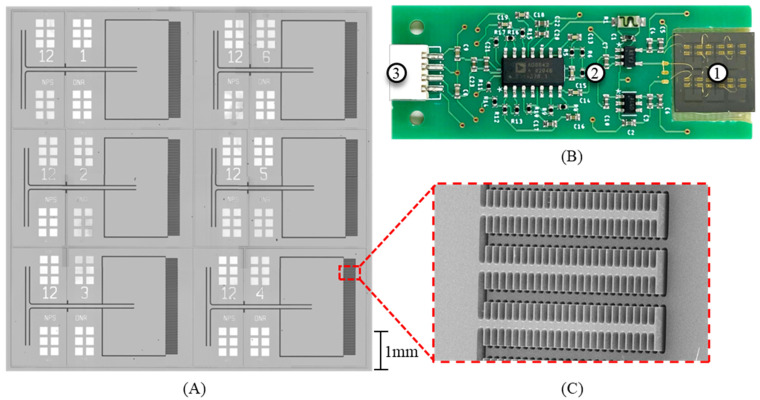
Multi-resonant acoustic sensor. (**A**) Microscope image of V12 sensor; (**B**) image of MEMS sensor mounted in PCB; (1) multi-resonant MEMS sensor, (2) capacitive readout circuitry, and (3) wire connections for power and readout; (**C**) SEM image of fishbone-patterned comb fingers. Note that residual stress on wings from fabrication causes wings to bend slightly, lifting wing comb fingers while at rest, approximately halfway up from substrate comb fingers.

Figure 3A shows a top-down picture of the sensor (version V12). A picture of the sensor mounted into a PCB is shown in Figure 3B. A scanning electron microscope (SEM) image of the fishbone-style comb fingers is shown in Figure 3C. Table 1 shows some of the key dimensional parameters of the sensor. The resonant frequency of each wing was set via the bridge length. The wing dimensions and torsional leg dimensions were maintained wing to wing.

### 2.3. Analytical Modeling

At resonance, each wing acts like a driven, damped harmonic oscillator. The sensor was limited to frequencies where only the first mode was excited. In this mode, we can think of a single wing as a mass-loaded spring system with two stiffnesses to consider: the bending of the beam and the twisting of the torsional legs. The analytical model discussed here follows from the analytical model presented in [16], with modifications to account for the twisting of the torsional legs.

**Figure 4:**
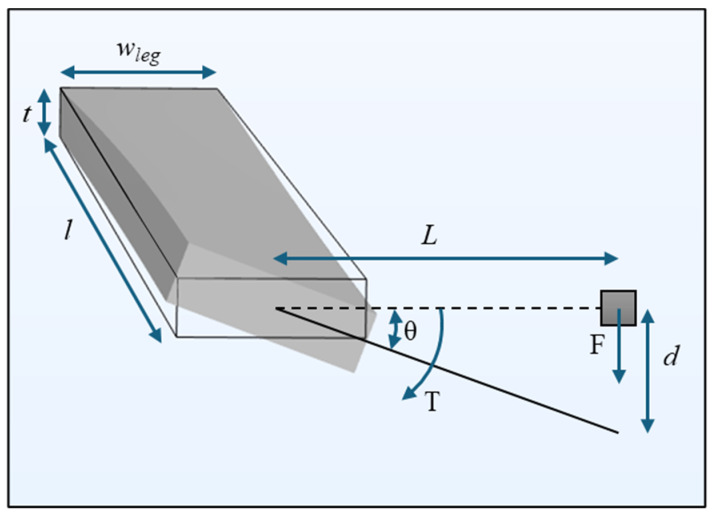
Relating torsional stiffness (*K*_*t*_) to flexural stiffness (*k*_*leg*_).

The wing is modeled as an undamped, simple harmonic oscillator with three springs. Two springs are in parallel (each torsional leg). Those springs are in series with the third spring (the bending of the bridge). The overall stiffness of the wing is given by

(1) $$k_{wing}={\left(\frac{1}{2k_{leg}}+\frac{1}{k_{bridge}}\right)}^{-1},$$

where *k_leg_* is the stiffness of a torsional leg, and *k_bridge_* is the stiffness of the bridge, which can be determined by the standard equation for a flexural beam [24]:

(2) $$k_{brige}=\frac{Ewt^{3}}{4L^{3}}\;,$$

where *E* is Young’s modulus of silicon. The parameters *w*, *t*, and *L* are the width, thickness, and length of the bridge, respectively. To determine the stiffness of the legs, first, the torsional stiffness, *K_t_*, must be established based on the physical properties of the torsional legs [24], which is determined by

(3) $$J_{leg}=G*\frac{w_{leg}t^{3}}{16}\left[\frac{16}{3}-3.36\frac{t}{w_{leg}}\left(1-\frac{t^{4}}{12{w_{leg}}^{4}}\right)\right]\;,$$

(4) $$K_{t}=\frac{J}{l}=\frac{T}{\theta }\;,$$

where *J_leg_* is the torsional rigidity of a single torsional leg, G is the shear modulus of the silicon, and the parameters *w_leg_*, *t*, and *l* are the width, thickness, and length of the torsional leg, respectively. Note that for this wing design, the thickness is consistent across the entire wing. T is the applied torque to the beam and *θ* is the twist angle at the end of the leg. Figure 4 shows a diagram representing how the torsional stiffness of the torsional legs relates to flexural stiffness. This allows the effects of the torsional legs and bridge to be combined as shown in (1).

*K_t_* can be related to *k_leg_* based on the twisting angle and applied torque from the force applied to the wing by the acoustic wave as follows:

(5) $$F=k_{leg}d=k_{leg}L*\tan\left(\theta \right),$$

(6) $$T=F*L=K_{t}\theta .$$

Combining (5) and (6) yields

(7) $$k_{leg}=\frac{K_{t}\theta }{L^{2}\tan\left(\theta \right)}.$$

However, for a small *θ*, tan (*θ*) ≈ *θ*. Therefore,

(8) $$k_{leg}=\frac{K_{t}}{L^{2}}\;.$$

The mass of the wing is approximated by an effective point mass, *m_eff_*, located at the end of the bridge. The moment of inertia of the point mass is equivalent to the moment of inertia of the wing. This technique is discussed in more detail in [16]. Neglecting damping effects, the resonant frequency, f_0_, of the wing can be modeled as follows:

(9) $$f_{0}=\frac{1}{2\pi }\sqrt{\frac{k_{wing}}{m_{eff}}}\;.$$

To include damping, this analytic model modifies the Sader [25] method to determine the resonant frequency and quality factor of a cantilever beam vibrating in a surrounding fluid. The Sader method is agnostic to the cross-sectional shape of the beam, but it assumes that the cross-section is constant across the length of the beam. The effective width of the beam, *b*, is determined based on its cross-sectional shape. The presented wing design does not meet this assumption. Therefore, the performance of previous wing designs was used to modify the method to determine *b*. The value of *b* is determined based on the widths of bridge and wing using the following formula:

(10) $$b=w_{wing}-0.016*\left(\frac{{w_{wing}}^{2}}{w}\right)\;.$$

The quality factor, *Q*, can be computed using

(11) $$Q=\frac{\frac{4\mu }{\pi \rho b^{2}}+\Gamma_{r}}{\Gamma_{i}}\;,$$

where *μ* is the dynamic viscosity, and *ρ* is the density of the fluid. Γ*_r_* and Γ*_i_* are the real and imaginary parts of the hydrodynamic function detailed in [25].

The modification of Sader’s method is discussed in more detail in [16]. The analytical model slightly underestimates the measured resonant frequency. The average modeled resonant frequency is 2% lower than the average measured resonant frequency for all wings. However, the analytical model underdamps the system with respect to the quality factor. The average modeled quality factor is approximately 1.8 times larger than the average measured quality factor. A detailed comparison of the analytical modeling, computer simulations, and measured sensor responses is provided in Section 4.

For an MEMS acoustic sensor of this type operating in air, only including damping from the modified Sader method is sufficient. However, if the sensor is operating in a more viscous fluid (e.g., water, silicone oil), additional damping effects such as Couette flow in the gaps between the wing and substrate and the capacitive comb fingers must be considered.

### 2.4. Finite Element Modeling

FE modeling of the sensor was conducted using COMSOL Multiphysics version 6.1 modeling software. The FE models were based on similar models to those described in [16]. Each wing in the sensor was modeled independently to determine its resonance frequency, response to driving frequency (i.e., wing displacement and phase), and directionality.

The device layer was modeled as anisotropic silicon, with the elasticity matrix aligned to the crystalline structure of the silicon. The sensor was enclosed within a sphere of air with standard properties from the COMSOL material library. A shell of air with perfectly matched layer properties was included around the sphere to prevent acoustic reflections. To reduce computational time, the FE model was bisected along the centerline of the sensor, and symmetry boundary conditions were applied along the bisection. Previously, similar FE models were used in the development of single-resonant MEMS sensors [16]. Based on the measured performance of those sensors, this FE model was updated to include an additional damping (drag) force so that the modeled behavior better matched the measured sensor performance.

**Figure 5:**
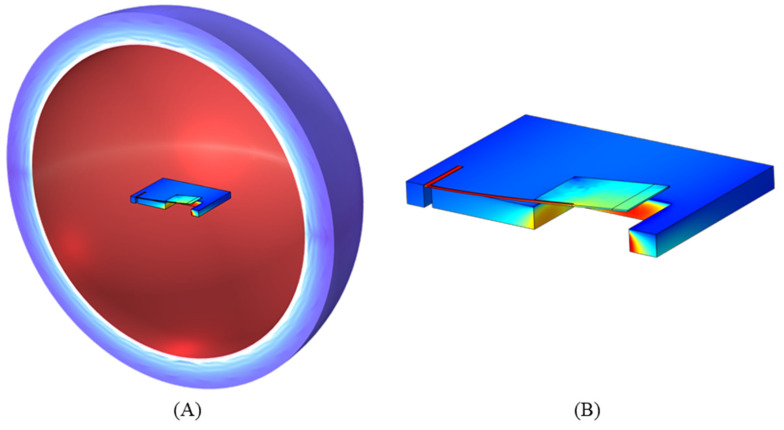
FE model images of single wing. (**A**) Depiction of the FE model. Wing located in center of sphere of air. (**B**) Single wing in bending mode. The FE model consists of only half the model and sphere or air. Symmetry conditions are applied along the bisection to account for the entire sensor.

**Figure 6:**
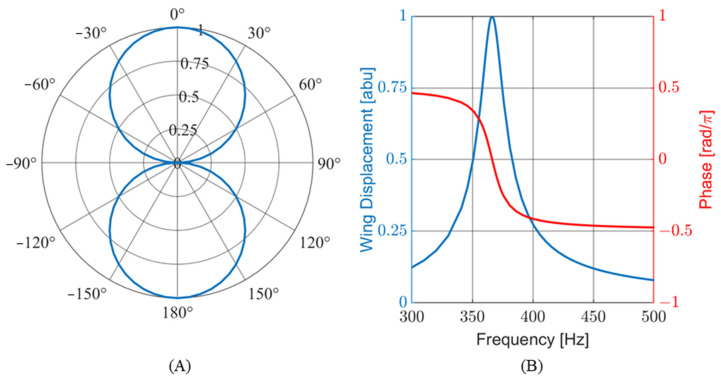
An FE model of wing behavior. (**A**) The modeled wing directionality matches an ideal cosine-like shape. (**B**) The Frequency response of a single wing showing normalized displacement (blue) and phase (red). A single wing behaves like a harmonic oscillator in terms of resonance.

The FE model used a free tetrahedral meshing for the sensor and surrounding sphere of air. The perfectly matched layer shell of air surrounding the sphere was meshed using a swept mesh method. Figure 5A shows a depiction of the device suspended in the sphere of air and surrounding a shell of air. Figure 5B shows a zoomed-in view of a single half-wing in the bending vibration mode. The solid mechanics module was used to set fixed constraints, boundary loads on the wings, and symmetry conditions. The pressure acoustic module was used to apply a plane wave pressure field to the sensor. The plane wave direction of propagation was adjusted with a parametric sweep to model the acoustic source rotating around the sensor to obtain the directionality pattern, as seen in Figure 6A. The simulation shows a cosine-like response, as expected. The frequency of the acoustic wave was adjusted with a separate parametric sweep to measure the displacement and phase response of the sensor, as seen in Figure 6B. The results show a harmonic oscillator behavior near resonance. An arbitrary phase offset was applied so that the phase equals zero at resonance. This offset was applied to match the algorithms used for DOA estimation.

The bending mode is the lowest-frequency resonant mode of the sensor design. An eigenfrequency analysis was conducted to determine the frequency of the second major resonant mode of the sensor. That mode consists primarily of the wing rocking back and forth laterally, pivoted at the point where the bridge meets the wing. The second mode for wing 1 is approximately 3018 Hz. Its deflection magnitude is approximately 15% that of the first resonant mode. This is outside of the range of interest and was filtered out by the electronics readout.

The FE model’s quality factor was determined by calculating the magnitude of wing displacement with respect to the frequency:

(12) $$Q=\frac{f_{0}}{f_{h}-f_{l}}$$

where *f*_0_ is the resonant frequency, and *f_h_* and *f_l_* are the upper and lower bounds of the frequencies, where the displacement magnitude is 70.7% of the maximum. The FE modeling results are compared with measured results in more detail in Section 4. However, the average modeled quality factor agrees with the measured quality factors within 0.6%. The average modeled resonant frequencies agree with the measured values within 0.9%. This demonstrates that FE modeling is an effective tool for sensor design.

## 3. Experimental Methods

### 3.1. Mechanical Sensitivity

**Figure 7:**
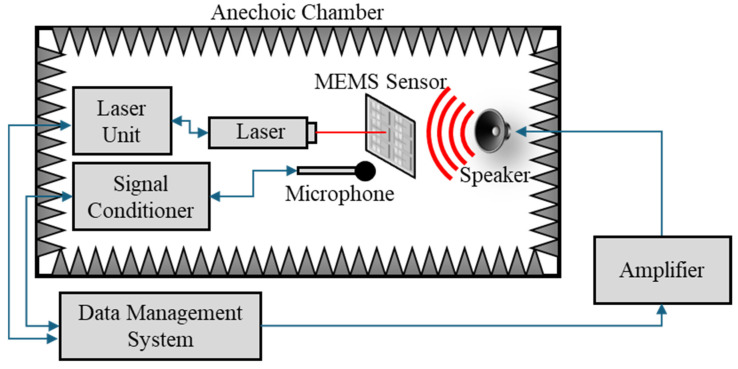
Laser vibrometry experimental setup.

Prior to cementing the sensor into the PCB and wire-bonding it to the capacitive readout circuit, the mechanical sensitivity was measured via laser vibrometry utilizing a Polytech data management system (DMS) computer, OFV-5000 controller, and an OFV-534 laser unit. Data collection was conducted in an anechoic chamber. The sensor was held in place in the path of a laser beam so that the beam terminated at the wing center line, near the far edge of a wing, just before the comb fingers. The DMS generated an audio signal (250 to 510 Hz frequency sweep) that was sent through a Techron 5507 amplifier to a JBL 7-inch speaker, which faced the sensor. The DMS measured the deflection of the sensor wing. The acoustic pressure was measured with a Piezotronics Model 378A21 reference microphone. The microphone signal was sent through a Piezotronics Model 482C signal conditioner to the DMS. The DMS would calculate the average deflection amplitude per acoustic pressure (mechanical sensitivity) of the wing, as a function of frequency, over the course of five frequency sweeps. Once the mechanical sensitivity of a given wing was measured, the sensor would be repositioned so that a different wing was moved into the path of the laser, and the process was repeated for each wing in the sensor. Figure 7 shows the experimental setup of the laser vibrometry.

### 3.2. Electrical Characterization in Air

After laser vibrometry measurements were taken, the sensor was cemented into the host PCB and wire-bonded to the capacitive readout circuit for directionality and frequency response measurements. The sensor was mounted on a precision turntable (B&K Model 5960) in an anechoic chamber, with a stationary speaker (7-inch JBL cone speaker) pointed at the sensor. Rotating the sensor changed the DOA at which the acoustic wave was incident upon the sensor. The MEMS sensor was connected to a control box, which provided power to the sensor and distributed the output to other devices. A calibrated reference microphone (Piezotronics Model 378A21) was mounted near the MEMS sensor. The signal from the microphone was sent to a signal conditioner (Piezotronics Model 482C). The outputs of the microphone and MEMS sensor were read by separate Zurich Instruments multifunction lock-in amplifiers (MFLIs).

**Figure 8:**
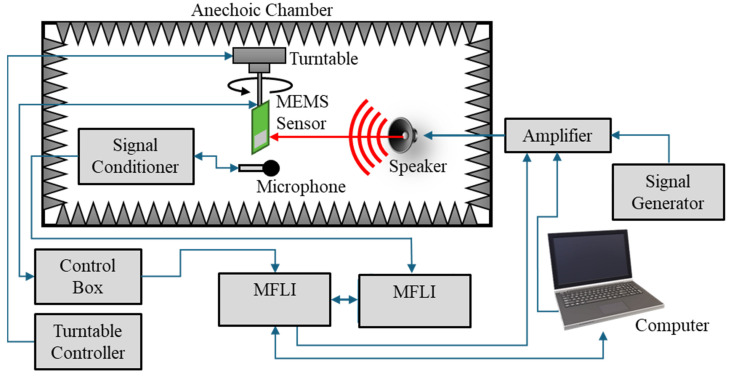
Experimental setup for sensor characterization (frequency response, directionality).

The MFLIs and a signal generator (Agilent 33220A) were used to produce various sounds (e.g., steady tones, white noise, frequency sweeps) to characterize the MEMS sensor. Signals from the MFLI and signal generator were sent to an amplifier (Techron 5507) and then to the speaker in the anechoic chamber. Figure 8 shows the experimental layout to determine the frequency response, directionality, and SNR of the MEMS sensor.

To determine the SNR, the MEMS sensor was mounted in an anechoic chamber. All electrical and acoustic equipment and noise sources were secured in the chamber, except for the MEMS sensor. The output of the sensor was read by an MFLI, which measured the noise spectral density over a bandwidth of 0 to approximately 12 kHz. To distinguish the electronic noise of the sensor circuitry from the mechanical noise of the MEMS sensor chip, two sets of noise spectral density measurements were taken. One set was for an unmodified sensor. The second set of data was with the wings glued in place to prevent their vibration, which removed the mechanical noise from the system.

### 3.3. Underwater Electrical Characterization

Underwater sensor characterization was conducted at the Naval Transducer Evaluation Center (TRANSDEC), a six-million-gallon, anechoic pool operated by the US Navy, to perform a wide range of underwater sensor characterizations.

**Figure 9:**
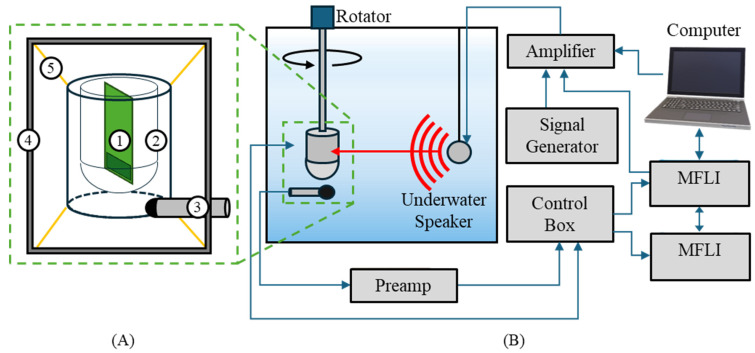
Underwater experimental setup. (**A**) Detailed diagram of the underwater sensor: (1) MEMS sensor, (2) air-tight sensor housing, (3) omnidirectional hydrophone, (4) mounting frame, and (5) elastic bands connecting housing to mounting frame. (**B**) Diagram of underwater experimental setup.

The MEMS sensor was enclosed in an air-filled, water-tight housing, as shown in Figure 9A. The sensor and housing were nearly neutrally buoyant. In this condition, the acoustic wave causes the sensor housing to vibrate, and the MEMS sensor acts as an inertial sensor, detecting the vibration of the housing rather than the acoustic wave directly. A similar experiment was described in [16].

The MEMS sensor and an omnidirectional reference hydrophone (B&K Type 8103) were mounted 6 feet deep on a pole with a motorized rotation mechanism. An underwater speaker (Electro Voice UW30) was suspended 6 m deep and 2 m away from the sensor. The output of the MEMS sensor was sent to a similar control box, discussed in Section 3.2. The output of the reference hydrophone was sent to a preamplifier (Stanford Research Systems SR560) and then to the control box. The control box directed the MEMS sensor and hydrophone outputs to the MFLIs for data collection. Acoustic signals were produced by either an MFLI, signal generator (Keysight 33500B), or computer. These signals were sent through an amplifier to the underwater speaker.

The characterization consisted of frequency response and directionality measurements. These measurements were conducted in a similar manner to those performed for the sensor in air, as discussed in Section 3.2. Figure 9B shows the experimental setup for data collected at TRANSDEC.

Additional frequency response measurements were taken in a water-filled standing wave tube (SWT). The sensor was mounted on the end of a pole, facing an underwater speaker (Electro Voice UW30) on the bottom of the SWT. The SWT produces a flat standing wave front at the sensor location. The SWT experimental setup was similar to the TRANSDEC setup, with an MFLI supplying a frequency sweep signal through an amplifier to the underwater speaker. The sensor output was then directed to the control box and then to the MFLI. A similar experimental setup using an SWT was discussed in [16].

## 4. Experimental Results

### 4.1. Frequency Response

**Figure 10:**
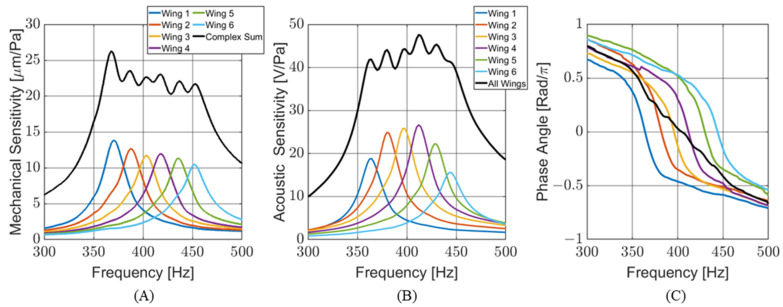
Measured sensor response: (**A**) mechanical sensitivity measured via laser vibrometry. Overall sensor response is calculated. (**B**) Acoustic sensitivity measured from output of capacitive sensing circuit. (**C**) Phase associated with acoustic sensitivity. Overall sensor response is measured for (**B**,**C**).

The mechanical sensitivities of the individual wings on the MEMS sensor were measured via laser vibrometry by measuring the deflection of the wing with respect to the applied acoustic pressure. Figure 10A shows the mechanical sensitivity of each wing individually. The complex sum of the wing sensitivities was calculated and plotted to predict an effective mechanical sensitivity of the entire sensor. The measured resonant frequencies and quality factors of the measured mechanical sensitivity are consistent with the FE models.

The acoustic sensitivity (output voltage per applied acoustic pressure) of the MEMS sensor was measured with the sensor cemented into the PCB with the capacitive readout circuit. The acoustic sensitivity is comparable to the mechanical sensitivity of the MEMS sensor. Figure 10B shows the sensitivity for each wing individually, as well as the entire sensor when all the wings, connected in parallel, were being read by the capacitive sensing circuit. The maximum sensitivity of the MEMS sensor was measured at 47.6 V/Pa (33.6 dB re 1 V/Pa). Figure 10C shows the phase response of the individual wings and their combination. Each wing behaves like its own harmonic oscillator. As predicted by the FE models, when the outputs of all the wings are combined, the phase response becomes more complex than those of single wings.

**Figure 11:**
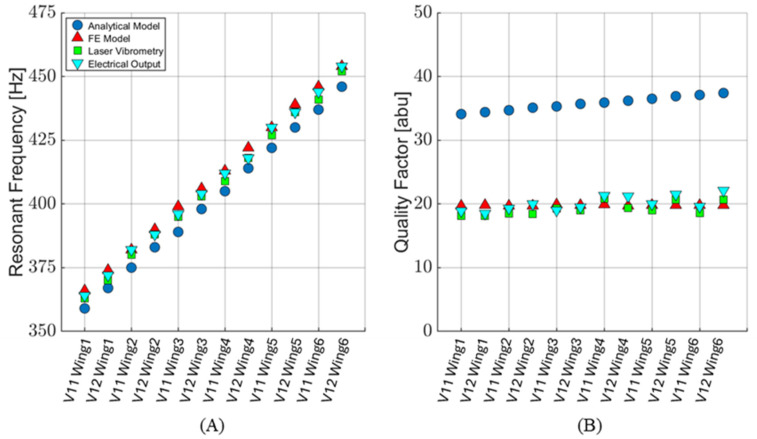
Comparison of analytical and FE models with measured results for sensor versions V11 and V12. (**A**) Modeled and measured resonant frequency. (**B**) Modeled and measured quality factor. The analytical model overestimates the quality factor of the sensor.

Table 2 shows the modeled and measured resonant frequencies and quality factors of individual wings for each sensor design, while Figure 11 presents these data graphically. The average percent difference in resonant frequencies between the FE model and measured electrical output is 0.43%. The quality factors agree within 0.59%. This shows that the FE model is an excellent predictor of the sensor’s frequency response. When comparing the laser vibrometry and electrical sensor performance, the average resonance frequencies agree to within 0.36%, and the average quality factors agree to within 4%. This suggests that any electrical damping effects created when applying a voltage across the MEMS sensor are not significant.

**Figure 12:**
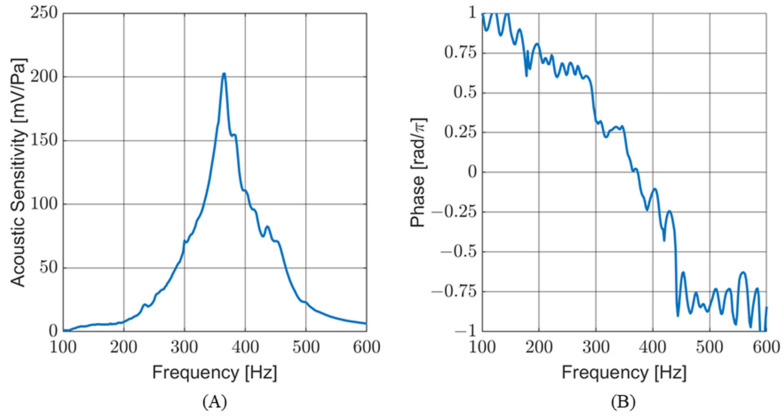
Underwater frequency response of the sensor. (**A**) Sensitivity of the sensor in SWT. (**B**) Phase response of the sensor at TRANSDEC.

The acoustic sensitivity was measured underwater, with the sensor mounted in an air-filled, water-tight housing mounted in an SWT. Figure 12A shows the sensitivity response of the sensor with respect to frequency. Figure 12B shows the phase response of the sensor, measured at the TRANSDEC facility. The wavy nature of the phase response is due, in part, to acoustic reflections and interference patterns generated in the pool during the frequency sweep. As expected, the frequency response of the sensor in an air-filled underwater housing is comparable to its response in air.

### 4.2. Directionality

**Figure 13:**
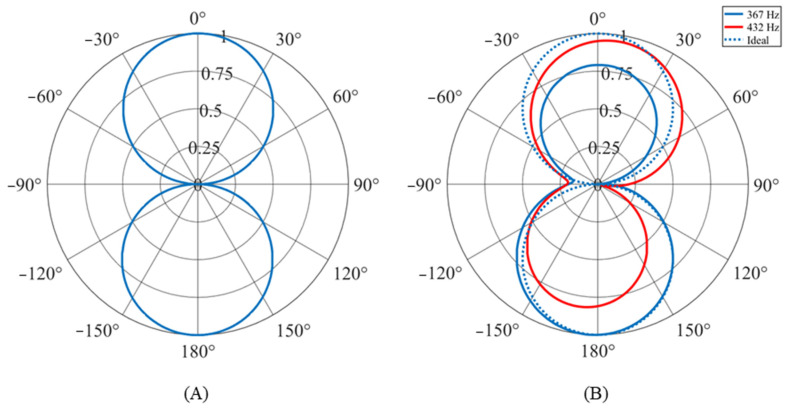
Directionality pattern of MEMS sensor: (**A**) in air and (**B**) under water.

Ideally, the sensors produce a cosine-like directionality pattern. Figure 13A shows the directionality of the sensor operating in air with a 429 Hz acoustic stimulus. The directionality very closely matches the ideal cosine-like shape. This directionality was consistent for all frequencies within the target bandwidth of the sensor (300 to 500 Hz). However, this is not the case when the sensor is operating under water.

Figure 13B shows the directionality patterns for the sensor while stimulated at two different frequencies. The solid blue line shows the directionality at 367 Hz and the red line at 432 Hz. A dotted blue line shows the ideal cosine-like directionality for comparison. While only two patterns are shown, they represent the varying directionality patterns measured over the bandwidth of the sensor. All patterns are pseudo-cosine-like (opposing lobes pointing towards 0 degrees and 180 degrees) with significant deviations from the ideal pattern: lobe size, lobe angle (lobe does not point directly at 0 degrees), and failure to go to zero at +/− 90 degrees. This inconsistent directionality is likely due to both the underwater acoustic environment where the data were collected and the sensor housing. Further investigation is needed to positively identify the causes.

### 4.3. Signal-to-Noise Ratio

**Figure 14:**
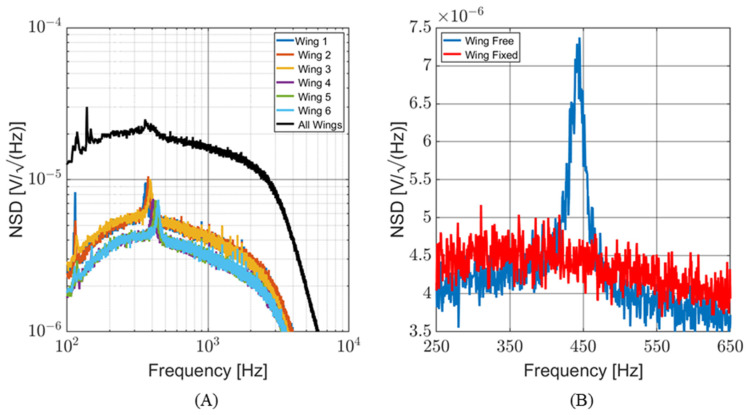
Noise spectral density of the MEMS sensor. (**A**) NSD of individual wings and the entire sensor. (**B**) Comparison of single wing while free to vibrate (blue line) and while fixed (red line). This demonstrates the mechanical contribution to the overall NSD.

Determining the SNR is critical to understanding the capabilities of this sensor. The noise spectral density (NSD) of the sensor (both individual wings and the entire sensor) was measured in an anechoic chamber, with all possible acoustic and electrical noise sources secured. The NSD data represent the mechanical and electronic noise of the MEMS sensor and associated readout circuitry. Figure 14A shows the NSD of each individual wing and the entire sensor (with all wings bonded to the readout circuit). The readout circuit has a bandwidth of 100 Hz to 3 kHz. The peaks in the NSD of individual wings correspond to their resonant frequencies. To isolate the mechanical and electrical portions of the NSD of a single wing, measurements were taken with the wing free to vibrate and again with the wing fixed (glued in place). The NSD curves with fixed wings closely match the curves with free wings, except for these resonant peaks. Figure 14B shows the NSD of a single wing, focusing on its resonance. The fixed wing’s NSD curve closely matches that of the free wing except for the resonant peak.

**Figure 15:**
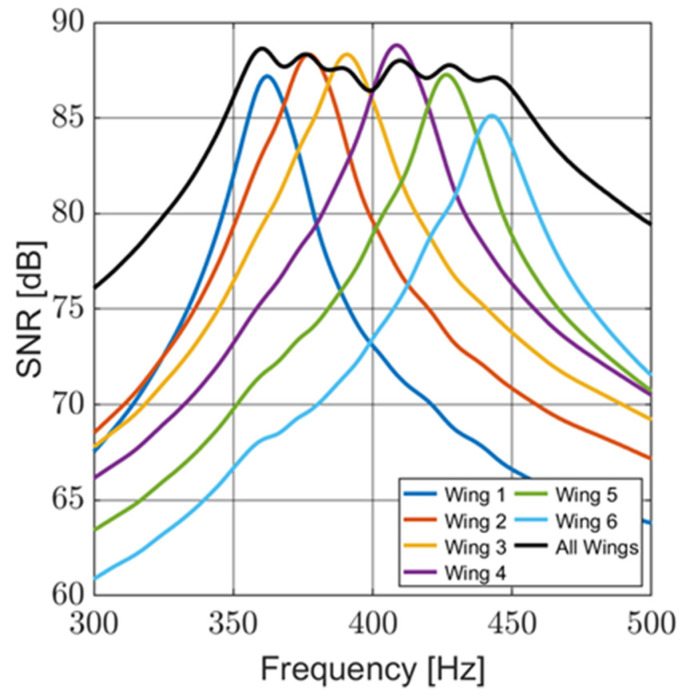
Signal-to-noise ratio of the sensor (individual wings and the entire sensor). The sensor demonstrates a high SNR over its design bandwidth.

The NSD data were used to calculate the noise level of the sensor over the bandwidth of the sensor circuitry (100 Hz to 3 kHz) and over the design bandwidth of the sensor (300 to 500 Hz). The acoustic sensitivity data were used to determine the signal level, at 1 Pa, with respect to frequency. Figure 15 shows the SNR for individual wings and the entire sensor, with the noise level based on the bandwidth of the sensor circuitry. The maximum SNR of the sensor over the circuit bandwidth is 88.6 dB, and over the design bandwidth, it is 97.4 dB. The sensor maintains a high SNR over the design bandwidth of the sensor.

## 5. Discussion and Conclusions

A multi-resonant, MEMS acoustic sensor was designed using analytical and FE modeling techniques. The sensor was characterized in air both mechanically (using laser vibrometry) and electrically to determine the directionality, frequency response, and SNR of the sensor. Additionally, the sensor was characterized underwater while contained in an air-filled housing. The sensor improves upon previous designs by broadening the effective bandwidth while maintaining a high SNR and cosine-like directionality.

### 5.1. Sensor Characterization

As seen in Figure 15, this sensor provides a very high SNR over a 200 Hz bandwidth. This is a significant improvement when compared to other resonant acoustic sensors. It was demonstrated in [21] for vibrating wing sensors operating at resonance that the SNR is proportional to the square root of the quality factor, tying a high SNR to narrow bandwidths. This multi-resonant design overcomes that limitation by maintaining a comparable SNR with nearly 4.5 times the bandwidth.

The sensor displays the same cosine-like directionality as other vibrating wing sensors. Deviations from the ideal directionality, which were observed for the underwater configuration of the sensor, were also seen in underwater version of previous designs [16].

### 5.2. Comparison with Similar Sensors

Table 3 compares the multi-resonant sensor’s SNR and effective bandwidth (based on the full width and half max of the resonant peak) performance. It shows the improved performance of the multi-resonant sensor when compared to similar resonant MEMS acoustic sensors.

### 5.3. Improving Sensor Designs

This versatile multi-resonant sensor can be scaled to different frequency ranges. The pass band can be expanded, and the size of the device can be reduced. Several techniques, commonly used in MEMS devices, can be applied to change the stiffness of the bridges and torsional beams, as well as the mass of the paddles, allowing for adjusting the spectral response of the sensor as desired while preserving a small size. The detection and localization of quiet sources with specific acoustic signatures such as sniper fire, multi-rotor small UAVs (drones), single- or multi-tone communication, or sonar signals when used underwater, etc., can be achieved. A combination of such sensors can be used to make acoustic vector sensors to provide full 3D coverage (azimuth and elevation). These applications are of particular interest to Defense and law enforcement.

Another interesting aspect of this approach is that if the readout mechanism is changed from capacitive comb fingers to piezoelectric films, which can be achieved without adding complexity to the sensor, this sensor can easily become a mechanical energy harvester [26,27]. Moreover, by broadening the resonant response, as demonstrated in this manuscript, or tuning the response to desired bands, a very efficient and flexible harvester can be designed.

## Figures and Tables

**Table 1:** Key sensor design dimensions.

Wing Width	Wing Length	Wing Thickness
2500 μm	1600 μm	25 μm
**Design Freq** **(V11 Wing 1)**	**Bridge Length** **(V11 Wing 1)**	**Torsional Leg** **Length**
366 Hz	2900 μm	1000 μm

**Table 2:** Resonant frequency and quality factor comparison of modeled and measured values.

Version V11 Resonant Frequency [Hz]/Quality Factor												
Data Source	Wing 1		Wing 2		Wing 3		Wing 4		Wing 5		Wing 6	
Analytical Model	359	34.1	375	34.7	389	35.3	405	35.9	422	36.5	437	37.1
Finite Element Model	366	19.7	382	19.7	399	19.9	413	19.9	430	19.8	446	19.8
Laser Vibrometry	363	18.1	380	18.5	395	19.3	409	20.8	427	19.0	441	18.6
Electrical Output	364	18.9	382	19.3	396	19.0	412	21.3	430	20.0	444	19.6
**Version V12 Resonant Frequency [Hz]/Quality Factor**												
**Data Source**	**Wing 1**		**Wing 2**		**Wing 3**		**Wing 4**		**Wing 5**		**Wing 6**	
Analytical Model	367	34.4	383	35.1	398	35.7	414	36.2	430	36.9	446	37.4
Finite Element Model	374	19.8	390	19.7	406	19.8	422	19.7	439	19.8	454	19.8
Laser Vibrometry	370	18.1	388	18.4	403	19.0	418	19.4	436	20.7	452	20.7
Electrical Output	372	18.5	388	20.0	404	19.5	418	21.2	436	21.5	454	22.1

**Table 3:** Sensor performance comparison.

Sensor	Sensitivity	SNR [dB]	Bandwidth
Multi-Resonant	48 V/Pa	88.6 (97.4) ^1^	300 Hz–500 Hz
Double-Wing Design [16]	59 V/Pa	88 (102) ^1^	658 Hz–684 Hz
Dual-Band Design [21]	13 V/Pa	91 ^1^	650 Hz–725 Hz ^2^
Double-Wing [9]	3.45 mV/Pa	68.5	Not Discussed

^1^ Noise based on sensor resonance bandwidth instead of bandpass of circuit. ^2^ Approximate values.

## Data Availability

The data that support the findings of this study are available from the corresponding author upon reasonable request.
